# Assessment of the Impacts of Land Use Changes on Nonpoint Source Pollution Inputs Upstream of the Three Gorges Reservoir

**DOI:** 10.1155/2014/526240

**Published:** 2014-04-07

**Authors:** Huicai Yang, Guoqiang Wang, Yan Yang, Baolin Xue, Binbin Wu

**Affiliations:** ^1^College of Water Sciences, Beijing Normal University, Beijing 100875, China; ^2^United Faculty of Agriculture, Gifu University, 1-1 Yanagido, Gifu 501-1193, Japan; ^3^State Key Laboratory of Vegetation and Environmental Change, Institute of Botany, Chinese Academy of Sciences, Beijing 100093, China

## Abstract

In recent years, land use upstream of the Three Gorges Reservoir (TGR) has changed significantly because of the TGR project. In this study, the Soil and Water Assessment Tool (SWAT) model was examined for its ability to assess relationships between land use changes and nonpoint pollutant indexes upstream of the TGR. Results indicated that the SWAT model, calibrated with the adjusted parameters, could successfully reproduce the nonpoint indexes at the water quality monitoring sites in the two rivers. The different land use change types were shown to be sensitive to nonpoint pollutants in the study area. The land use change type from upland to water was the strongest influence on changes in total nitrogen and total phosphorus. An empirical regression equation between nonpoint indexes and different land use change types was developed for the study area by partial least squares regression (PLSR) as follows: *Y* = *b*_0_ + ∑_*i*=1_^*m*^ *b*_*i*_ *X*_*i*_. This regression equation was useful for evaluating the influence of land use change types on changes in nonpoint pollutants over a long time period. The results from this study may be useful for the TGR management and may help to reduce nonpoint pollutant loads into downstream water bodies.

## 1. Introduction 

The Three Gorges Reservoir (TGR) area is of significant strategic importance to the Yangtze River Basin and the sustainable development of China. With rapid economic development in recent years, increasing pollution from point and nonpoint sources has led to considerable degradation of water resources. Furthermore, the water environment has increased in complexity since the completion of the Three Gorges Dam [1]. Thus, reliable information on water quality and pollution sources in this region is important for effective water management. Because of the reservoir construction, many people in this area have been displaced and have had to move, which has resulted in continuously changing land use patterns since 2000. These changes will influence runoff patterns and also the water quality in this area [2, 3].

Many studies have reported that land use change can influence nutrient generation and transport in surface flow. Osborne and Wiley [4] examined the relationships between nitrogen (N) and phosphorus (P) concentrations in streams and land use/land cover (LULC) patterns in the Salt Fork watershed, Illinois. Yong and Chen [5] examined the hydrological effects of land use in Ohio and discovered that there was a significant relationship between LULC types and surface water quality, especially for N and P. In the TGR area, Zhang et al. [6] used a relatively long-term dataset of biotic and abiotic water quality variables from the Daning River and, by using correlation and redundancy analysis (RDA), showed that the total nitrogen (TN)/total phosphorus (TP) ratio was the main factor influencing phytoplankton growth in this area. Ding et al. [7] used the Soil and Water Assessment Tool (SWAT) model in the same basin to simulate surface runoff water quality and found that agricultural land was the dominant source of nonpoint pollution.

For many years, hydrological models have proved useful for simulating and predicting water resources and nonpoint pollutants. The Soil and Water Assessment Tool (SWAT) was developed specifically to simulate water quantity and quality in large complex catchments over long time periods. This popular tool can simulate hydrological processes and water quality of surface, soil, and underground water. In this study, the two small basins of the Dong and Puli Rivers, which are located in the area upstream of the TGR, were evaluated using SWAT. Since the commencement of water storage in the TGR in 2003, land use has changed significantly in these basins [8]. In this contribution, we discuss relationships between land use change and water quality, based on detailed land use change types in the study area. To explain water quality, many previous studies have emphasized the amounts or proportions of certain land use types, such as urban and agricultural land uses. Xiao and Ji [9] used statistical analyses to quantify the relationship between landscape metrics and surface water quality using remote sensing and water quality data. There have been fewer studies on the effects of land use change types on nutrient transport in this area, so a combined approach should be applied in the TGR for analyzing relationships between land use change types and changes in nutrients.

In the study area, partial least squares regression (PLSR) has already proved to be a useful method for analyzing the relationship between runoff generation and land use change [8]. The main aim of our study was to quantify the impact of land use change types on nutrient changes in the region upstream of the TGR. SWAT was used to simulate hydrological and nutrient processes in the Pengxi River basin, located upstream of the TGR. Land use maps derived from Landsat Thematic Mapper (TM) images acquired in 2000 and 2010 were used to analyze basin land use changes. Data for nutrient generation and transport in the river channel was used to examine the relationship between land use change types and corresponding nutrients, and PLSR and GIS landscape pattern analysis were used to develop correlations between land use change and nonpoint pollutant indexes. Results were analyzed to determine which land use change types had most influence on nonpoint pollutant indexes in the region upstream of the TGR.

## 2. Study Area Description

**Figure 1:**
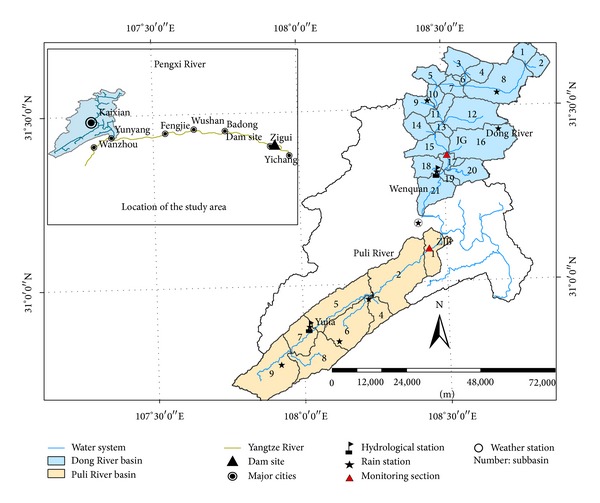
Location of the study area.

The Three Gorges Reservoir, in the middle reaches of the Yangtze River in China, is one of the largest reservoirs in the world. It was built to harness hydropower and mitigate floods and droughts in the middle and lower reaches of the Yangtze and connected lake basins and tributaries. The Pengxi River (30°50′–31°42′N, 107°56′–108°54′E) is the largest subtributary of the Yangtze River, located north of the Three Gorges Reservoir area. The Pengxi River basin covers an area of about 5,172.5 km^2^ [2]. Average annual precipitation over the basin is 1,100–1,500 mm, and the average annual discharge is about 3.41 billion m^3^. Because of discharge data availability, we focused on two major tributaries of the Pengxi River basin, the Dong and Puli Rivers (Figure 1). About 221,500 immigrants have settled along the two rivers since the TGR project was implemented. Land use patterns of the upstream basin of the TGR were relatively stable through the 1980s, but they have changed significantly since the 1990s because of socioeconomic development of neighboring cities and the TGR project construction. Land use changes have been especially significant in the period from 2003, since the TGR began to store water.

## 3. Methodology

### 3.1. Soil and Water Assessment Tool (SWAT) Description

SWAT is a temporally continuous, physically based hydrological model and was used to represent hydrological and water quality processes [10]. The model subdivides a watershed into subbasins connected by a stream network and further delineates hydrologic response units (HRUs) consisting of unique combinations of land cover and soils in each subbasin. The hydrological routines within SWAT account for snowfall and snowmelt, vadose zone processes (i.e., infiltration, evaporation, plant uptake, lateral flows, and percolation), and groundwater flows [11].

SWAT simulates N and P cycles through five different pools of N comprising two inorganic forms and three organic forms, and six different pools of P, including three inorganic forms and three organic forms in soil [12]. Both N and P cycles include mineralization, decomposition, and immobilization processes. Organic N and P transport with sediment is estimated using a loading function [13, 14]. Daily organic N and P runoff losses are calculated by loading functions based on the concentrations of these elements in the top soil layer and in eroded sediment and an enrichment ratio. The nitrate (NO_*x*_-N) concentration in mobile water is calculated and multiplied with the volume of mobile water to estimate total NO_*x*_-N lost from the soil layer. Soluble P transported in runoff is estimated using the P concentration in the top soil layer, the runoff volume, and a soil P partitioning coefficient [15].

The Nash-Sutcliffe (Nash) coefficient of efficiency [16] and the coefficient of determination (*R* ^2^) were used as the objective function for optimizing the model performance. The N-S coefficient is defined as

(1) $$\begin{array}{c} \text{Nash}=1-\frac{\sum_{i=1}^{n}{\left(Q_{oi}-Q_{si}\right)}^{2}}{\sum_{i=1}^{n}{\left(Q_{oi}-\bar{Q_{o}}\right)}^{2}}, \end{array}$$

where *Q*_*oi*_ (m^3^ s^−1^) is the observed data, *Q*_*si*_ (m^3^ s^−1^) is the simulated data, *n* is the total number of records for comparison, and $\bar{Q_{o}}$ (m^3^ s^−1^) is the mean value of the data observed over the simulation period. *R* ^2^ is the proportion of variation explained by fitting a regression line and is seen as a measure of the strength of the linear relationship between simulated and observed data.

### 3.2. Partial Least Squares Regression (PLSR)

Partial least squares regression (PLSR) method is an effective technique for finding the relationship between the properties of a molecule and its structure. In mathematical terms, PLSR relates a matrix *Y* of dependent variables to a matrix *X* of molecular structure descriptors, that is, a latent variable approach to modeling the covariance structures in these two spaces [17]. PLSR allows for the analysis of data with strong correlations in the predictor variables and when the number of training samples is far smaller than that of predictor variables [18]. The most outstanding advantage is applicability to the situation in which there is high multicollinearity between predictors. PLSR can achieve a higher accuracy with a smaller number of predictor variables, relative to multiple linear regression and principal component analysis [19]. During cross validation, the optimal number of components for the model is determined based on the predicted residual sum of squares (PRESS) [20]. The minimum PRESS is normally used to indicate the optimal number of components [21]. Once the optimal component number is identified, regression coefficients are extracted. Only those variables with significant contribution (i.e., regression coefficient larger than 0.05) to the response variable are selected for constructing the best model and their regression coefficients are recalculated in that model [19].

The relationship between the land use change types and nonpoint indexes was obtained using the PLSR method which was implemented using the Minitab 15 statistical software [22].

The general formula can be expressed as follows:

(2) $$\begin{array}{c} Y=b_{0}+\overset{m}{\underset{i=1}{\sum }}b_{i}X_{i}. \end{array}$$

In this study, the percentage of the subbasin area where the land use changed between 2000 and 2010 were used for the model fitting. Here, *X*_*i*_  (*i* = 1, 2,…, 7) and *Y* represent percentages of eight land use change types and the nonpoint pollutant indexes, respectively. *m* is the number of variables, *b*_*i*_ is the regression coefficient, and *b*_0_ is the constant.

### 3.3. Data Preparation

**Figure 2:**
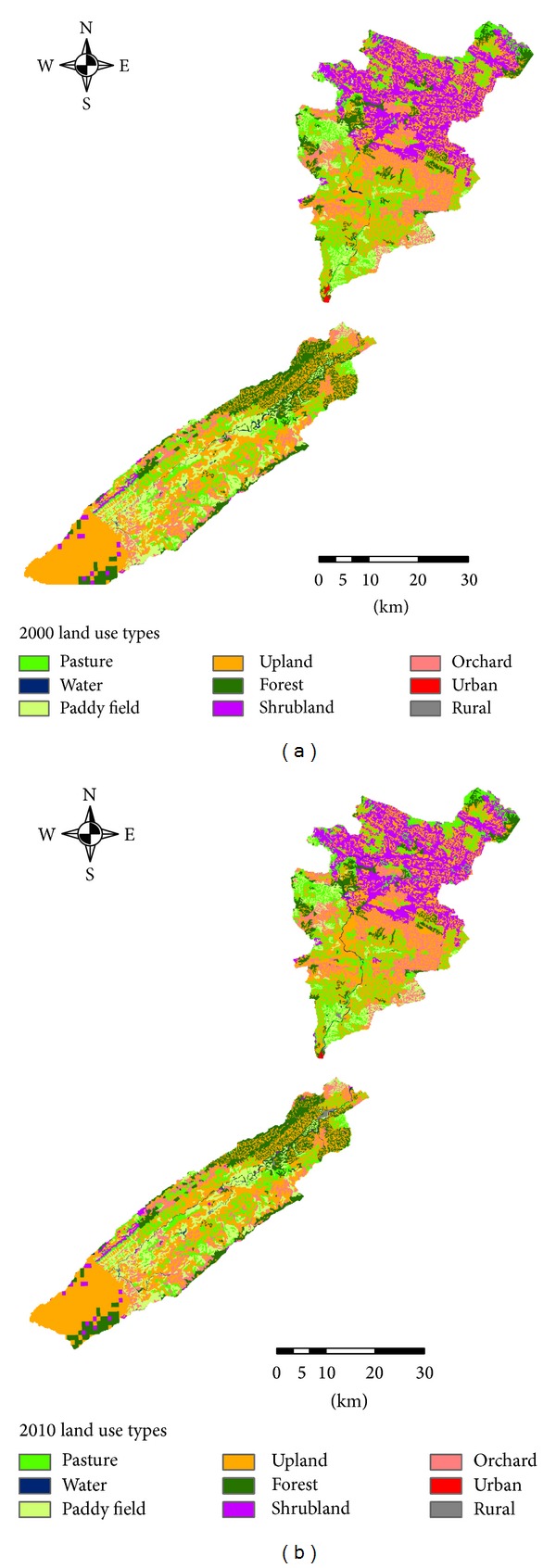
Land use maps of the Pengxi River basin in (a) 2000 and (b) 2010.

**Figure 3:**
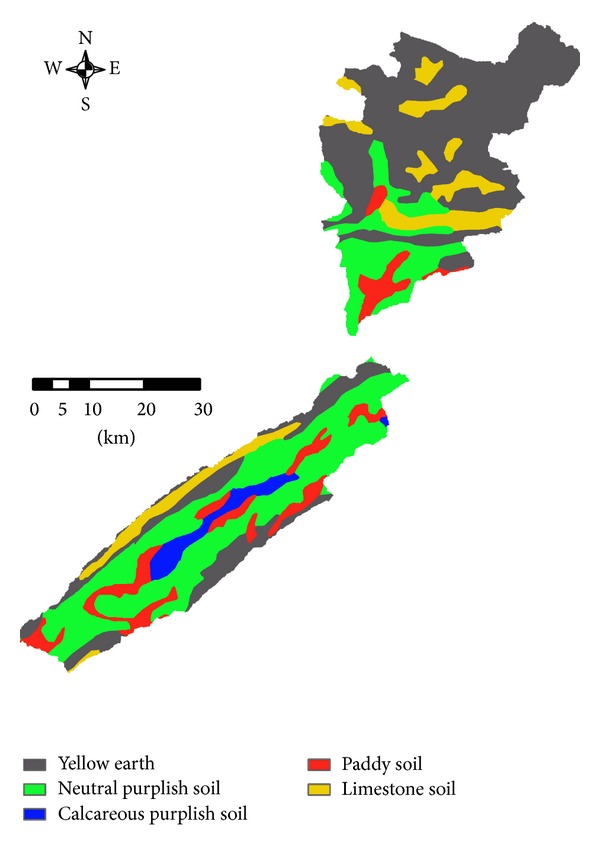
Soil maps of the Pengxi River basin.

DEM data at a 90 m resolution were used to generate the topographic information needed for the SWAT model, such as river channels, channel width, slope, and subbasins. Land use maps and soil maps were used to extract the HRUs, the basic unit for calculating parameters in the SWAT model. Land use maps were developed from Landsat 5 Thematic Mapper (TM) data (Figure 2), and 9 land use types were defined (Table 1). Five soil types were identified from the Chinese National 1 : 1,000,000 scale soil map (Figure 3). The original DEM digital spatial data, land use map, soil type map, and river drainage map were converted to the same spatial resolution. All input spatial data were processed into the Albers equivalent conical projection system, as required for the SWAT simulation.

The meteorological, hydrological, and water quality monitoring data used to construct the SWAT model are shown in Table 2. Precipitation, minimum and maximum air temperature, and wind speed data, collected from meteorological stations within the study area (Figure 1), were used as the daily climate inputs for SWAT. Additional climate variables, such as solar radiation and dew-point temperature, were produced by a weather generator using values from the nearest standardized weather station. Daily discharge data were collected from gauging stations in the two basins (Figure 1). Water quality monitoring data were collected from the Jinguan (JG) and Zhaojia Bridge (ZJB) monitoring stations in the Dong and Puli Rivers, respectively.

## 4. Result Analysis and Discussion

### 4.1. Sensitivity Analysis

In this study, eighteen parameters were identified as sensitive and were tested for the Pengxi River basin and are shown in Table 3, along with the sensitivity order from the sensitivity analysis. Water quality was most sensitive to the denitrification threshold water content (*SDNCO*). This parameter controls the amount of NO_*x*_-N removed from the surface layer in runoff relative to the amount removed via percolation, and it plays a critical role in the TN simulation processes. Other sensitive parameters were the P percolation coefficient (*PPERCO*), residue decomposition coefficient (*RSDCO*), soil P partitioning coefficient (*PHOSKD*), local rate constant for organic P mineralization (*BC4*), P enrichment ratio (*ERORGP*), a constant for biological oxidation of ammonia nitrogen (*BC1*), the contribution of the groundwater soluble P concentration to streamflow from the subbasin (*GWSOLP*), a soil erodibility factor (USLE_K), and the coefficient rate for humus active organic nutrients mineralization (CMN). After this analysis, a daily time step was used for calibration and validation, and all parameters were modified during model calibration.

### 4.2. Model Calibration and Validation

Based on the rainfall station distribution, the natural river network, and basin topography, the Pengxi River basin was divided into 30 subbasins, as shown in Figure 1. Using land use, soil properties, and slope data, these basins were further divided into 181 HRUs. Initial values of the model parameters were obtained from input maps and the database.

**Figure 4:**
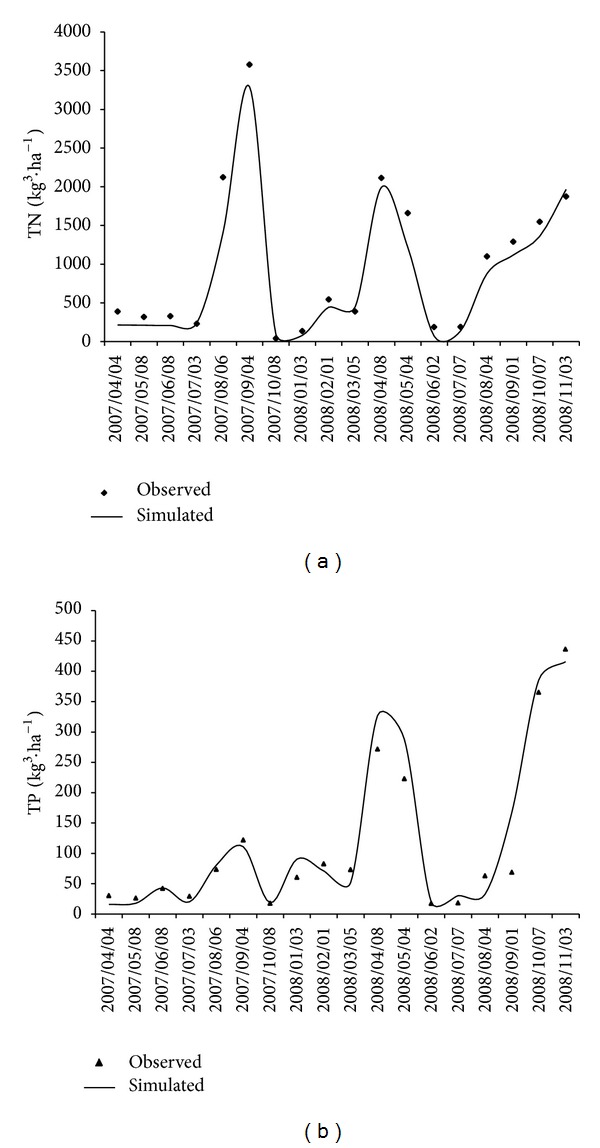
Time series of simulated and observed daily TN and TP from 2007 to 2009 at the JG station in the Dong basin.

To estimate the impact of land use change on nonpoint pollution, the hydrological processes of the SWAT model were calibrated and validated using land use data from 2000 and daily discharge data from the Wenquan and Yujia stations, located at the outlets of the Dong and Puli Rivers, respectively. Water quality processes (N and P cycles) were then calibrated and validated. An automatic parameter estimation procedure, SWAT-CUP (CUP stands for calibration and uncertainty procedures), was used to estimate parameter values for runoff and water quality simulation. To evaluate land use change effects on nonpoint pollution between 2000 and 2010, land use data from 2010 was used in the model after it had been calibrated and validated with 2000 land use data, with all other parameters remaining the same. The results of calibration and validation for the hydrological processes are given in Table 4. The N and P cycles in the Dong and Puli River basins were calibrated using the observed data from 2007 and 2008, collected from the JG and ZJB water quality monitoring stations, and validation was based on observed data from 2009 (Table 5). Results of the TN and TP calibration and validation show that all of the *E*_ns_ values were over 0.55 and *R* ^2^ values were more than 0.7.  Figure 4 shows the TN and TP scatterplot and time series for the validation phases at the JG and ZJB water monitoring stations for the Dong and Puli River basins and demonstrates that the SWAT model was able to satisfactorily simulate temporal variations in daily TN and TP.

### 4.3. Land Use Changes

**Figure 5:**
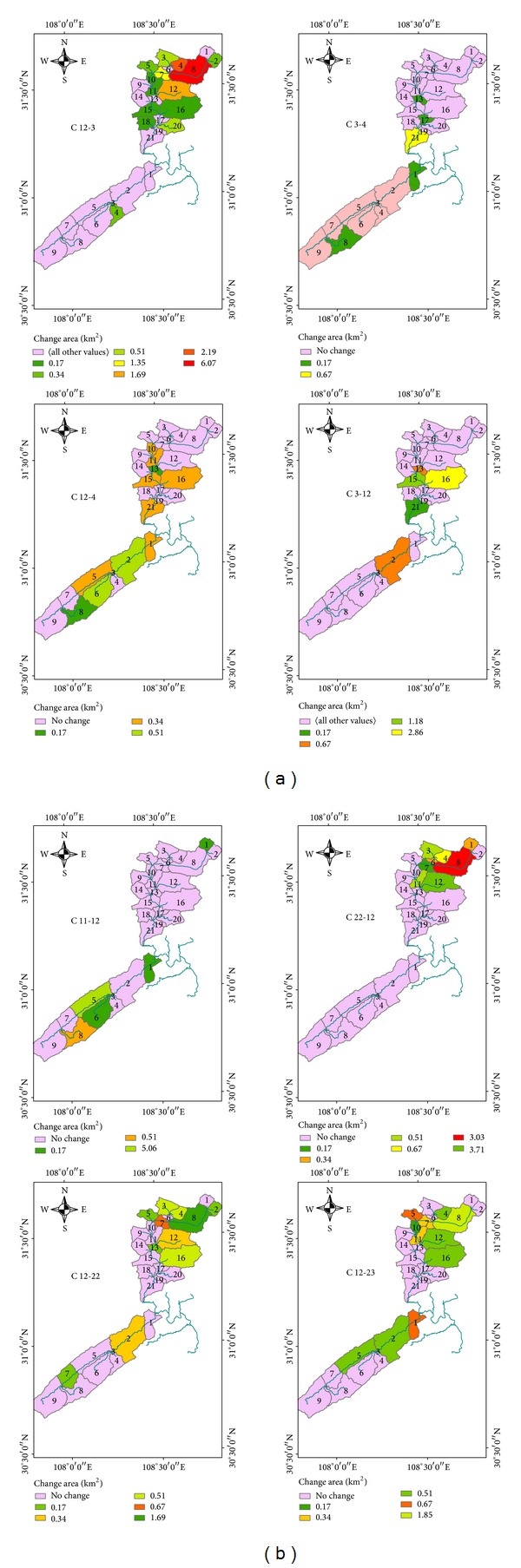
Spatial variation of land use conversion from 2000 to 2010. C12–3 upland to pasture; C3-4 pasture to water; C12–4 upland to water; C3–12 pasture to upland; C11-12 paddy field to upland; C22–12 shrubland to upland; C12–22 upland to shrubland; C12–23 upland to orchard.

Land use maps were developed from Landsat 5 TM data. To measure the land use composition within the 30 selected subbasins of the Pengxi River basin, we used land use maps in raster format from a geographical information system (GIS). The original land use map distinguished 15 land use types. We reclassified the land uses into 9 categories: paddy field, upland, forest, shrubland, orchard, pasture, water, urban, and rural (Table 1). In a previous study, we found that the main land use types in the Pengxi River basin were upland, paddy field, pasture, and forest [8]. We did more detailed research on land use change as part of this previous study. We calculated the transfer matrix from 2000 to 2010 for the Pengxi River basin and allocated the 72 change types between the 9 land use types and then identified the 8 top most common land use types based on the area of the main land use change types in the study area (Figure 5). The category axis represents the codes for each land use change type. Preliminary analysis indicated that the main land use change types during this period included pasture conversion to upland, upland to orchard, upland to pasture, pasture to water, upland to shrubland, pasture to water, shrubland to upland, and paddy field to upland. The total area of the 8 land use types is 105.62 km^2^, which accounts for 50% of the whole area of land use change. Most of the 8 land use change types were in the 5 main land use types.

To analyze the characteristics of the spatial distribution of the main land use change types in detail, we identified the distributed subbasins of each land use change type (Figure 5). We found that every land use change type tended to be clustered in certain areas. Analysis indicated that upland to pasture (C12–3) areas occurred in 13 subbasins in Dong River basin, accounting for 17.41% of the Dong River basin, and 1 subbasin in the Puli River basin. Pasture to water (C3-4) areas were mainly distributed in 5 subbasins in the downstream basins of the Dong and Puli Rivers. Upland to water areas occurred in 11 subbasins and were evenly distributed in the middle and lower subbasins of Dong and Puli River basins. The area of pasture to upland change type was the greatest of all of the change types, with a change area of 17.18 km^2^, but it only involved 5 subbasins, which were generally distributed in the middle and lower reaches of Dong and Puli Rivers. Conversion from paddy field to upland areas generally occurred in the upper and middle subbasins of the Puli River basin. The changes between shrubland and upland (C12–22 and C22–12) occurred mainly in the upper and middle subbasins of Dong River basin, with more changes from upland to shrubland. Upland to orchard conversions were ranked second, with a change area of 17.08 km^2^, occurring in 12 subbasins. These were generally distributed in the upper and middle subbasins of the Dong and Puli River basins.

These changes were clearly a consequence of the TGR. The TGR, the world's largest hydropower project, has created a reservoir 600 km long with a surface area of 1060 km^2^ along the Yangtze valley between Yichang and Chongqing. It was constructed from 1993 to 2009. Its water level is 175 m above sea level, and its total storage capacity is 39.3 billion m^3^. In June 2003, the water level reached 135 m above sea level, and, with its continued rise, 1.13 million inhabitants had to be relocated, many to the upstream river basins. From 2000 to 2004, further 96 000 residents from the region along the upper reaches of the TGR were relocated [23]. This placed significant additional pressure on land use in the local area, which was already intensively disturbed. Overall, land use in basins upstream of the TGR has been strongly influenced by socioeconomic development and the TGR project.

### 4.4. Impact of Land Use Changes

**Figure 6:**
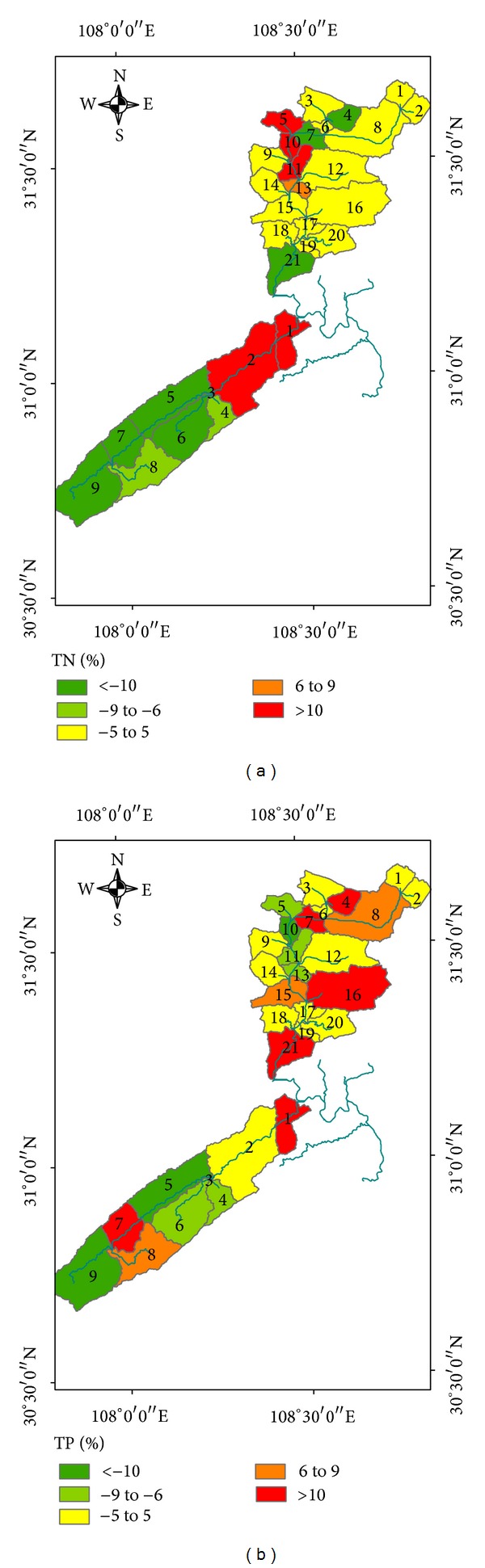
Spatial variation of water quality from 2000 to 2010 (a) percent change in TN; (b) percent change in TP.

**Figure 7:**
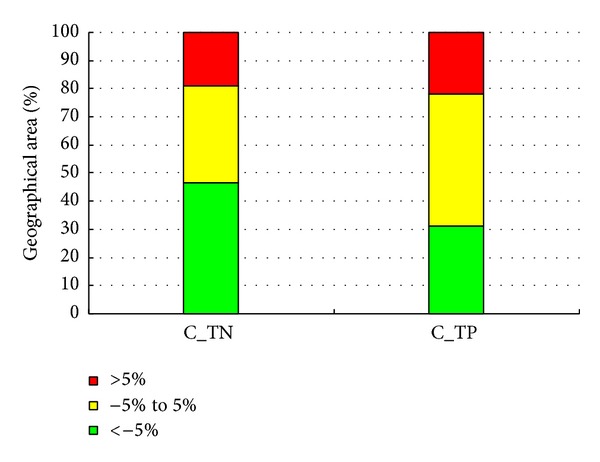
Percentage of geographical area under positive high, modest, or negative high classes.

Water quality changes at the subbasin level are presented in Figure 6. In general, the basins can be divided into three major classes: (1) positive high: if the percentage change in nonpoint pollutant indexes is greater than or equal to 5% of the original value; (2) modest: if the percentage change in hydrological characteristics is between −5% and 5% of the original value; and (3) negative high: if the percentage change in hydrologic characteristics is less than or equal to −5% of the original value (Figure 7). This demonstrates that the percent change in TN is modest in the majority of the subbasins of Dong River basin, except in subbasins 5, 10, and 11, in all of which upland to orchard was the main land use change type. In addition, decreases in TN were mostly observed upstream and downstream, which may be due to conversion from upland to pasture. More than 70.3% of the area, mainly distributed in the upstream reaches of the Puli River basin, was classified as negative high for TN. The remaining downstream area was classified as positive high. The majority of the region experienced modest changes in TP, with about 21.7% of the area classified as positive high and 31.1% classified as negative high. The positive high region is mostly associated with conversion of upland to orchard and water, which increased TP production.

The objective of this section is to examine whether land use conversion can explain nonpoint pollutant indexes at the subbasin level. To estimate the percentage of land use conversion within each of the 30 subbasins (Figure 1), the 2000 and 2010 land use maps were overlain. The top 8 land use conversion classes were identified and the percentage of land use conversion within each of the 8 classes relative to the total subbasin area was calculated for all 30 subbasins. The strength of the relationship (correlation) between the percentage of land use conversion within a subbasin and two nonpoint pollutant indexes was assessed (TN and TP). As shown in Table 6, changes in nonpoint pollutant indexes were examined by considering the percentage change in a variable relative to the 2000 land use scenario value (P_N, P_P), as well as the absolute difference in the variable (D_N, D_P). Several significant correlations were found, even at the 0.01 level (Table 6), using the Spearman test. Differences can be found between the correlations as they were described as in the forms of absolute and percentage differences. However, there was a clear correlation between the percentage change area and the percentage change of all nonpoint pollutant indexes in land use conversion from upland to water. Meanwhile, the correlation coefficients were relatively high with few exceptions (e.g., typically higher than 0.6), such as the percentage change of TP in percentage change area of land use conversion code 5 (upland to pasture). Most of the significant correlations were observed for the changes from upland land use to pasture, water, and shrubland types. The percentage changes of TN and TP were positively correlated with land use conversion code 15 (upland to water) at significant levels. The reason for these changes was that the nitrogen and phosphorus enriched surface soils in the previous upland area release nitrogen and phosphorus to the water body directly after the land use change. Therefore, this conversion was found to be one factor that increased the TN and TP concentrations in the downstream area. The percentage change of TP was found to be significantly correlated with land use conversion codes 53 and 51 (upland to pasture and shrubland). And the negative correlation indicates that the TP flux decreased when land use changed from upland to pasture and shrubland. These correlations reveal that the upland land use is the most important source for both TN flux and TP flux in this region, while different land use change may increase or attenuate the release of the these pollutions. Meanwhile, the previous study has also shown that agricultural lands produced 20 times higher phosphorus as the forest lands and 154 times that of barren land use [5].

### 4.5. Quantitative Analysis of Land Use Effects on Nonpoint Pollutant Indexes

The purpose of establishing the relationships between water quality indexes and percent land use change was twofold: (1) to understand how water quality indexes respond to land use change and (2) to make new or improved estimations of the nonpoint pollutant indexes by applying the established empirical relationships to future conditions. Derived from the PLSR, standardized regressions of the nonpoint indexes are given.

The regression models with standardized regression coefficients for respective variables are listed in Table 7. The standardized coefficients can be used as indicators of the sign and magnitude of the control of predictor variables on the response variable.

**Figure 8:**
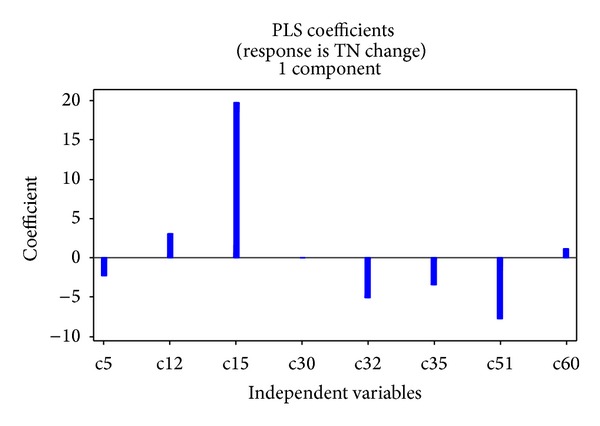
Coefficient map of TN PLRS results.

**Figure 9:**
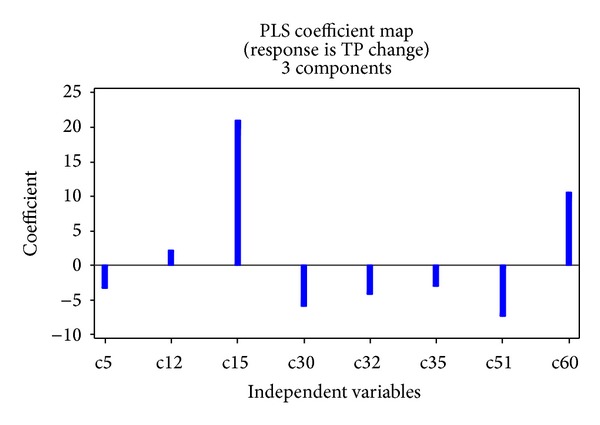
Coefficient map of TP PLRS results.

The optimal number of components for the best PLSR model, identified by using the minimum PRESS value, was 2. Eight predictor variables were included in the best model. Their standardized regression coefficients in the best model are listed in Table 7, and the largest three coefficients of land use change type were codes 15, 32, and 51, which indicates that they were the land use change types that influenced TN fluxes most (Figure 8). Land use change type codes 15, 51, and 60 play an important role in the TP fluxes (Figure 9). The fitted models are

(3) CTN=−2.21c5+3.07c12+19.76c15+0.03c30−5.05c32 −3.44c35−7.80c51+1.12c60+4.35CTP=−3.30c5+2.18c12+21.06c15−5.88c30−4.13c32 −3.06c35−7.42c51+10.58c60+2.25.

**Figure 10:**
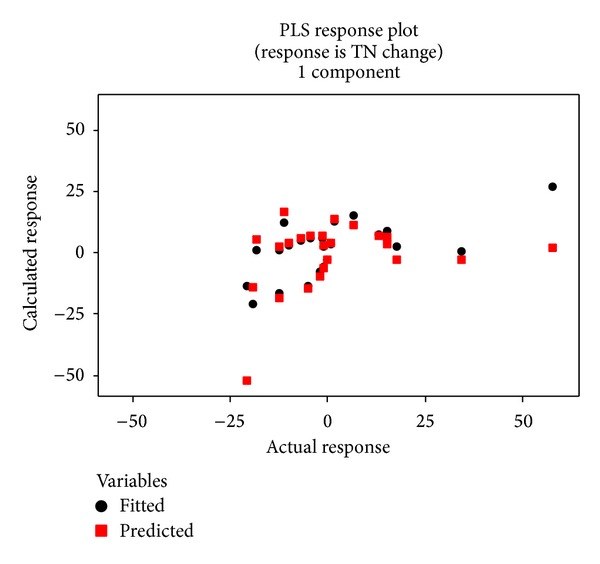
Scatter plot of the fitted and cross-validated data versus the actual TN changes.

**Figure 11:**
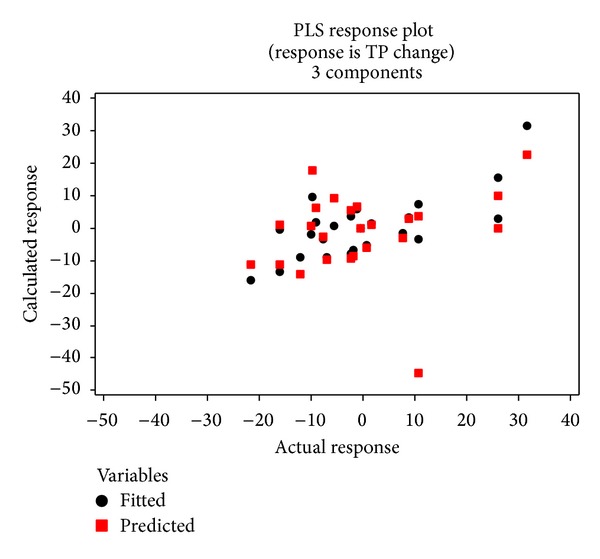
Scatter plot of the fitted and cross-validated data versus the actual TP changes.

The model is overall highly significant with a *P* value less than 0.001. The *R* ^2^ values are greater than 0.5, indicating that the model fits the data relatively well and that it can satisfactorily predict the data. The data prediction performance of the model can be also illustrated by plotting the fitted and predicted values (Figures 10 and 11). The points on the plot show a general linear pattern for the low data range but are widely scattered for the high data range, suggesting that the model is not very accurate for the high data range. The scatter for the high data range may be due to the land uses with higher areas of change making the land use change more complex.

The PLSR modeling result indicates that land use change type codes 12, 15, 30, and 60 have positive impacts on the nonpoint pollutant indexes, while codes 5, 32, 35, and 51 have negative impacts on the nonpoint pollutant indexes. The results for codes 32 and 35 are not as might have been expected, as two intertransformation types between upland and shrubland should have had opposite coefficients from the PLSR model; however they are both negative. This may be due to instability of the PLSR method and limitations of the sample data. For example, Shen et al. [24] found that nutrient simulations for forests showed higher uncertainty than those for grassland and plantation in the SWAT model, which may help explain the PLSR result. Correlation results suggest that code 32 should be positive, while code 35 should be negative.

Land use conversions from upland and pasture to water are mainly in the middle and lower reaches of the Dong and Puli River basins, where the most densely populated urban or rural areas are located. In these areas, water (including river, lake, reservoir, and pond) showed signs of pollution from human activity. In addition, large amounts of precipitation can cause polluted water to overflow into water bodies, thereby impacting negatively on nonpoint pollutant indexes; this may help explain the positive impact of land use conversion codes 12 and 15 on nonpoint pollutant indexes. Many studies have reported that agricultural land use contributes to degraded water quality in nearby aquatic systems, by altering the soil surface conditions, increasing the impervious area, and generating pollution [5, 25, 26]. Our results agree with previous findings that suggest that most of the water quality index model coefficients have negative signs when upland changes to other land use types (Tables 6 and 7). However, Lee et al. [27] reported that there was a fairly weak relationship between agricultural land use and water quality indexes, particularly for nutrients such as TN and TP. The degree to which agricultural land use has a negative influence on water quality depends on farming practices and geographic location [28, 29]. In our study area, upland is the primary agricultural land use, and, as previously discussed, upland cultivation differs from paddy field cultivation in many ways (e.g., [5, 25]). Paddy fields in the study area receive more intensive fertilizer applications than the upland cultivated areas. We therefore expected negative impacts of land use conversation code 32 on nonpoint pollutant indexes due to differences in fertilization between paddy fields and upland. For vegetation classified as shrubs, the dominant characteristic is that the shrubs or trees have a minimum height of 1.5 m and a dense understory [30]. Shrubland is usually made up of populations such as solanum deflexicarpum, birchleaf pear, myrsine stolonifera, pyracantha fortuneana, and fern and sometimes has been classified as a kind of forest [31, 32]. Generally, forest plant litter contains higher levels of organic nutrients than crop residue. Furthermore, litter biodegradability and breakdown is lower in forest soils than in crops [33] and herbaceous plants [34], meaning that land use changes from shrubland to upland lead to a negative impact on nonpoint pollutant indexes.

## 5. Conclusions

A distributed hydrological model was used to evaluate the effects of land use change on nonpoint pollution indexes (TN and TP). In this study, eighteen parameters were identified as sensitive and were tested for the Pengxi River basin. Using selected sensitive parameters, the SWAT model was calibrated and validated for the JG and ZJB water quality monitoring stations, located in the Dong and Puli Rivers, respectively. The model gave satisfactory results for TN and TP simulations at a daily time interval. The transfer matrix from 2000 to 2010 showed that there were 8 main land use change types, which, covering 105.62 km^2^, accounted for more than 50% of the total area of land use change. Most land use change was between upland, paddy field, pasture, shrubland, and water. Land use change between 2000 and 2010 was partly due to human activities, for example, the TGR. Spearman correlation results show that the change from upland to water had most effect on TN and TP. PLSR modeling of TN and TP also showed that the upland to water land use change plays a very important role in TN and TP changes, contributing more than one-third of importance to the TN and TP changes.

## Figures and Tables

**Table 1:** Land use type value and name.

Value	SWAT name	Land use type
11	AGRL	Paddy field
12	RICE	Upland
21	FRSD	Forest
22	RNGB	Shrubland
23	ORCD	Orchard
3	PAST	Pasture
4	WATR	Water
51	URBN	Urban
52	URML	Rural

**Table 2:** Main data sources.

Data	Data item	Station	Data period	Sources
Meteorological data	Maximum and minimum temperature,	Kaixian	2001–2010	State Meteorological Administration
	solar radiation,			
	sunshine percentage,			
	weed speed, and			
	relative humidity			
	Precipitation	Wenquan	2001–2010	
		Guanmian		
		Dajin		
		Yanshui		
		Yunyang		
		Hexing		
		Yujia		
		Nanmen		
		Qiaoting		
		Hexing		

Hydrological data	Discharge	Wenquan	2002–2010	Hydrological Statistical Yearbook
		Yujia	2001–2005, 2010	

Water quality data	TN and TP	Jinguan	2007–2009	Water quality monitoring section
		Zhaojia bridge	2007–2009	

**Table 3:** List of sensitive parameters calibrated and ranges and values of major parameters used.

Name	Description	Range		Optimum value	Sensitivity
		Min	Max		
SDNCO^1^	Denitrification threshold water content	0.908	0.987	0.974	0
PPERCO^2^	Phosphorus percolation coefficient (10 m^3^/Mg)	14.578	14.770	14.674	0.02
RSDCO^3rs^	Coefficient for mineralization of the residue fresh organic nutrients	0.052	0.086	0.070	0.14
PHOSKD^4^	Phosphorus soil partitioning coefficient	128.810	133.328	131.069	0.17
BC4^5^	Local rate constant for organic phosphorus mineralization at 20°PHC (day^−1^)	0.267	0.283	0.277	0.21
ERORGP^6^	Phosphorus enrichment ratio	1.913	1.929	1.928	0.23
BC1^7^	Rate constant for biological oxidation of ammonia nitrogen at 20°C (day^−1^)	0.116	0.128	0.121	0.23
GWSOLP^8^	Concentration of soluble phosphorus in groundwater contribution to streamflow from subbasin (ppm).	0.134	0.152	0.152	0.24
USLE_K^9^	Soil erodibility factor (0.013 metric ton m^2^ hr/(m^3^-metric ton cm))	5.752	6.963	6.600	0.25
CMN^10^	Rate coefficient for mineralization of the humus active organic nutrients	0.001	0.001	0.001	0.26
SHALLST^11^	Initial depth of water in the shallow aquifer (mm H_2_O)	2.773	2.992	2.989	0.42
USLE_C^12^	Minimum value for the cover and management factor	0.028	0.036	0.032	0.67
GW_DELAY^13^	Delay time for aquifer recharge (days)	1.584	1.670	1.608	0.71
SPEXP^14^	Exponent in sediment transport equation	1.270	1.270	1.270	0.81
ERORGN^15^	Organic nitrogen enrichment ratio	1.926	1.930	0.154	0.9
REVAPMN^16^	Threshold water level in shallow aquifer for revap or percolation to deep aquifer (mm H_2_O)	282.438	291.261	286.850	0.94
NPERCO^17^	Nitrate percolation coefficient	0.583	0.609	0.596	0.95
CH_K^18^	Effective hydraulic conductivity in main channel alluvium	54.074	55.028	54.551	0.98

^a^Superscripts of each parameter are their corresponding orders from the sensitivity analysis.

**Table 4:** Simulation results of runoff in the two basins.

Station	Simulation period	*E*_ns_	*r* ^2^
Wenquan station	Calibration for 2002–2006	0.94	0.94
	Validation for 2007–2010	0.98	0.99

Yujia station	Calibration for 2001–2002	0.80	0.81
	Validation for 2003–2005, 2010	0.93	0.87

**Table 5:** Simulation results of TN and TP nonpoint pollution in the two basins.

Simulation period	TN				TP			
	JG		ZJB		JG		ZJB	
	r^2^	E_ns_	r^2^	E_ns_	r^2^	E_ns_	r^2^	E_ns_
Calibration 2007-2008	0.85	0.6	0.76	0.74	0.83	0.55	0.74	0.64
Validation 2009	0.88	0.67	0.78	0.72	0.85	0.61	0.76	0.67

*JG is the water monitoring station of the Dong River basin, Jinguan; ZJB is the water monitoring station of the Puli River basin, Zhaojia bridge.

**Table 6:** Spearman correlation coefficients and probabilities (*P* value). Correlations between percentage of land use conversion within a subbasin and water quality variables. Bold coefficients indicate significant relationships.

2000	2010	Code	Area (km^2^)	P_N	P_P	D_N	D_P
Upland	Pasture	5	16.85	−0.47	−0.57*	−0.53	−0.65
				0.09	0.03	0.05	0.01
Pasture	Water	12	18.84	−0.21	−0.61	−0.15	−0.70
				0.73	0.28	0.81	0.19
Upland	Water	15	12.63	0.81**	0.69*	0.64*	0.46
				0.00	0.02	0.03	0.16
Pasture	Upland	30	17.18	0.32	0.65	0.45	0.56
				0.54	0.16	0.37	0.24
Paddy field	Upland	32	10.17	−0.47	−0.45	−0.29	−0.55
				0.43	0.45	0.64	0.34
Shrubland	Upland	35	10.70	0.08	0.85	0.08	0.21
				0.85	0.65	0.85	0.62
Upland	Shrubland	51	12.16	−0.45	−0.62*	−0.25	−0.52
				0.14	0.03	0.44	0.08
Upland	Orchard	60	17.08	0.13	0.02	0.11	0.04
				0.70	0.95	0.70	0.90

P_N: percent changes in total nitrogen %.

P_P: percent changes in total phosphorus %.

D_N: differences in total nitrogen (kg·ha-1).

D_P: differences in total phosphorus (kg·ha-1).

*n* = 23.

**P < 0.05.

*P < 0.01.

**Table 7:** Fitted models and model performance of TN and TP changes.

Standardized regression coefficients				
Land use change types			*C*_TN_	*C*_TP_
2000	2010	code		
Upland	Pasture	5	−0.17	−0.32
Pasture	Water	12	0.05	0.04
Upland	Water	15	0.33	0.45
Pasture	Upland	30	0.001	−0.31
Paddy field	Upland	32	−0.18	−0.19
Shrubland	Upland	35	−0.16	−0.18
Upland	Shrubland	51	−0.21	−0.26
Upland	Orchard	60	0.02	0.30

Model performance				
*n*			23	23
*F* statistic			13.26	7.24
*P* value			0.001	0.002
*R* ^2^			0.56	0.62
